# The Effects of Pharmacological Inhibition of Histone Deacetylase 3 (HDAC3) in Huntington’s Disease Mice

**DOI:** 10.1371/journal.pone.0152498

**Published:** 2016-03-31

**Authors:** Haiqun Jia, Ying Wang, Charles D. Morris, Vincent Jacques, Joel M. Gottesfeld, James R. Rusche, Elizabeth A. Thomas

**Affiliations:** 1 Department of Cellular and Molecular Neuroscience, The Scripps Research Institute, La Jolla, California, United States of America; 2 California Institute for Biomedical Research, La Jolla, California, United States of America; 3 Repligen Corporation, Waltham, Massachusetts, United States of America; 4 Department of Cell and Molecular Biology, The Scripps Research Institute, La Jolla, California, United States of America; Central Michigan University, UNITED STATES

## Abstract

An important epigenetic modification in Huntington’s disease (HD) research is histone acetylation, which is regulated by histone acetyltransferase and histone deacetylase (HDAC) enzymes. HDAC inhibitors have proven effective in HD model systems, and recent work is now focused on functional dissection of the individual HDAC enzymes in these effects. Histone deacetylase 3 (HDAC3), a member of the class I subfamily of HDACs, has previously been implicated in neuronal toxicity and huntingtin-induced cell death. Hence, we tested the effects of RGFP966 ((*E*)-N-(2-amino-4-fluorophenyl)-3-(1-cinnamyl-1*H*-pyrazol-4-yl)acrylamide), a benzamide-type HDAC inhibitor that selectively targets HDAC3, in the N171-82Q transgenic mouse model of HD. We found that RGFP966 at doses of 10 and 25 mg/kg improves motor deficits on rotarod and in open field exploration, accompanied by neuroprotective effects on striatal volume. In light of previous studies implicating HDAC3 in immune function, we measured gene expression changes for 84 immune-related genes elicited by RGFP966 using quantitative PCR arrays. RGFP966 treatment did not cause widespread changes in cytokine/chemokine gene expression patterns, but did significantly alter the striatal expression of macrophage migration inhibitory factor (*Mif)*, a hormone immune modulator associated with glial cell activation, in N171-82Q transgenic mice, but not WT mice. Accordingly, RGFP966-treated mice showed decreased glial fibrillary acidic protein (GFAP) immunoreactivity, a marker of astrocyte activation, in the striatum of N171-82Q transgenic mice compared to vehicle-treated mice. These findings suggest that the beneficial actions of HDAC3 inhibition could be related, in part, with lowered *Mif* levels and its associated downstream effects.

## Introduction

A growing body of literature has implicated histone deacetylase (HDAC) inhibitors as candidate drugs for the treatment of different neurological disorders, including Huntington’s disease (HD) [1, 2]. HD is an inherited, progressive neurodegenerative disorder characterized by chorea, movement dysfunction, cognitive impairment, and behavioral disturbances [3]. Although early studies utilizing broad-spectrum HDAC inhibitors showed beneficial effects in different HD mouse models [4], current ideology suggests that subtype-selective inhibitors may prove more beneficial for the treatment of neurological disorders, as these compounds might have fewer harmful side effects, caused by global inhibition of all HDAC proteins [5].

In mammals, there are 11 zinc-dependent HDAC proteins that are grouped into three classes. Class I HDACs are mainly localized to the nucleus and consist of HDAC1, HDAC2, HDAC3, and HDAC8. Class II HDACs, which can shuttle between the cytoplasm and nucleus, are subdivided into class IIa (HDAC4, HDAC5, HDAC7, and HDAC9) and class IIb (HDAC6 and HDAC10). HDAC11 is the sole member of class IV [6]. It is becoming clear that individual HDACs play distinct roles in mammalian physiology, and HDAC3 has been implicated in several functions associated with neurological disorders. Early studies implicated HDAC3 in neurotoxicity and cell death, where elevated *Hdac3* expression was found to promote the death of rat cerebellar granule and cortical neurons, but had no effect on the viability of primary kidney fibroblasts and HeLa cells [7, 8]. It was further shown that shRNA-mediated suppression of *Hdac3* expression protected against potassium deprivation-induced neuronal death [7]. These landmark findings indicated that HDAC3-induced toxicity is cell-selective and that neuronal cells are most vulnerable. Later studies by the same group demonstrated a specific link between HDAC3 and HD, where HDAC3 was shown to interact with the Htt protein [9]. HDAC3 has also been implicated in inflammatory gene expression pathways [10, 11]. A study investigating the role of HDAC3 in the induction of inflammatory genes in macrophages found that *Hdac3*-deficiency resulted in reduced expression of inflammation-related genes after stimulation by lipopolysaccharide [10]. In another study, HDAC3 was found to mediate allergic skin inflammation by regulating monocyte chemoattractant protein-1 [11]. Although these studies were conducted in peripheral tissues, the findings are pertinent to HD in light of emerging evidence implicating immune activation and inflammation in the pathogenic progression of this disorder [12–15].

We have previously studied benzamide-type HDAC inhibitors, which preferentially target HDAC1 and HDAC3 enzymes, for their potential therapeutic effects in HD. Our previous studies have shown that these compounds can improve huntingtin protein-elicited phenotypes in HD *Drosophila*, ST*Hdh*^Q111^ striatal cells and mouse models, and show low *in vitro* and *in vivo* toxicity [16–19]. However, given the above-mentioned literature implicating HDAC3 specifically in HD, in this study we further explored the effects of an HDAC3-selective inhibitor, RGFP966 ((*E*)-N-(2-amino-4-fluorophenyl)-3-(1-cinnamyl-1*H*-pyrazol-4-yl)acrylamide) [20], in HD transgenic mice to assess its effects on motor behavior and neuroprotection. Further, given that HDAC3 has been implicated in immune events, we explored potential immune-related effects elicited by HDAC3 inhibition in the brain in this mouse model.

## Materials and Methods

### Mice

All procedures were in strict accordance with the National Institutes of Health Guidelines for the Care and Use of Laboratory Animals and were approved by the Scripps Research Institute’s Institutional Animal Care and Use Committee. All procedures are designed to cause only momentary or no pain to the animals, although HD transgenic mice in this study may suffer mild distress due to disease progression (motor dysfunction & weight loss). Further details regarding the ethical use of experimental animals are provided in S1 Methods. A B6C3-Tg(HD82Gln)81Dbo/J (N171-HD82Q) line (Jackson Laboratories) has been maintained at The Scripps Research Institute by breeding male heterozygous N171-HD82Q mice with F1 hybrids of the same background. At the age of 3–4 weeks, mice were genotyped according to the Jackson Laboratories protocol to determine hemizygosity for the HD transgene. The CAG repeat lengths in these mice were verified by commercial genotyping (Laragen, Los Angeles, CA) and found to be 82 ± 1 CAGs for the major transgene species. The lifespan of the N171-82Q HD is ~18–20 weeks with HD-like symptoms beginning at 9–10 weeks of age. Groups of mice (N = 12–16 per genotype and drug treatment) were used for these studies.

### Drug treatment

RGFP966 was provided by Repligen Corporation (Waltham MA). N171-82Q transgenic mice were housed and maintained on a normal 12-h light/dark cycle with lights on at 6:00 a.m and free access to food and water. Mice were administered RGFP966 (10 or 25 mg/kg) for 10 weeks by S.C. injection (3 injections/week) beginning at 8 weeks of age. RGFP966 was dissolved with 75% polyethylene glycol 200/25% sodium acetate (6.25 mM); control mice received an equal volume of drug vehicle. Body weights were recorded twice per week. Mice were sacrificed at 18 weeks of age, 6 h after the final injection by overdose with isofluorane anesthesia. Brains were removed, and striata and cortex dissected out for gene expression assays or intracardially perfused with 4% paraformaldehyde.

### Motor behavioral assessments

#### Rotarod

An AccuRotor rotarod (AccuScan Instruments) was used to measure motor coordination and balance from mice at 12 weeks of age. Mice were tested on during the light phase of the 12-h light/dark cycle by using an accelerating rotation paradigm. Mice were placed on a rotarod accelerating from 0 to 40 RPM over 600 s and latency to fall was recorded. Mice were given four trials on four consecutive days. The baseline level of performance is recorded on day 1 of the 4-day test and is used to compare against performance on subsequent days.

#### Open Field Test

Open field exploration was measured in a square plexiglass chamber (27.3 cm x 27.3 cm x 20.3 cm) at 14 weeks of age (Med Associates Inc, St Albans, VT). Several behavioral parameters (Ambulatory Distance, Ambulatory Time, Stereotypic Time, Vertical Time, Vertical counts and Mean Velocity) were recorded during a 10 minute observation period. Statistical analyses for these motor tests were carried out using Two-way ANOVA using GraphPad software (GraphPad Prism, San Diego, CA).

### PCR arrays

Real-time qPCR analysis for the PCR arrays was performed using the RT^2^ SYBR green qPCR Master Mix (SABiosciences) on a StepOnePlus*™* Real-Time PCR System (Life Technologies) as described previously [21]. cDNA from groups of male mice (n = 4 per group) was tested using the Cytokines & Chemokines RT² Profiler PCR Array (Cat#: PAMM-150ZE), which contains 84 genes related to inflammatory pathways and the immune response (S1 Table). A set of 5 housekeeping genes (HKG) was used as internal controls for standardization between samples: *Gusb*, *Hprt*, *Hsp90ab1*, *Gapdh* and *Actb*. Results were analyzed using the PCR Array Data Analysis Web Portal (SABiosciences), followed by Benjami-Hochberg FDR correction.

### Real-time qPCR analysis

Real-time qPCR experiments were performed using the ABI StepOne Detection System (Applied Biosystems, Foster City, CA) as described previously [21]. Amplification was performed on a cDNA amount equivalent to 25 ng total RNA with 1 x SYBR^®^ Green universal PCR Master mix (Applied Biosystems) containing deoxyribonucleotide triphosphates, MgCl2, AmpliTaq Gold DNA polymerase, and forward and reverse primers. PCR reactions were performed on groups of n = 6 mice per condition. Specific primers for each sequence were designed using Primer Express 1.5 software and their specificity for binding to the desired sequences was searched against NCBI database (S2 Table). The amount of cDNA in each sample was calculated using SDS2.1 software by the comparative threshold cycle (Ct) method and expressed as 2exp(Ct) using hypoxanthine guanine phosphoribosyl transferase (*Hprt*) as an internal control. Statistical significance was determined using Student’s t tests (unpaired; two-tailed) (GraphPad Prism, San Diego, CA).

### Striatal volumetric analysis

To assess grey matter shrinkage in the brains of drug treated mice compared to vehicle, brain sections (25 μm) from vehicle and RGFP966-treated HD transgenic mice were stained with Richardson’s dye and images captured using Axiovision LE software. The total area of the striatum was assessed using sections 125 μm apart spanning the striatum from ~bregma, 1.18 to 0.38 mm. These brain sections were manually traced and volumes recorded using Image J software. On average, the volumes from n = 5–10 striata were averaged to obtain a single value per mouse. Statistical significance was determined by One-way ANOVA, with a Dunnett’s post-test (GraphPad Prism, San Diego, CA).

### Immunohistochemical analyses

Immunohistochemistry experiments were performed on free-floating brain sections (25 μm) from vehicle, and RGFP966-treated N171-82Q transgenic mice, as described previously [22], using an anti-GFAP antibody (Abcam; 1:500 dilution) or anti-Huntingtin, EM-48 (Abcam; 1:500 dilution). The immunoreaction was detected with Vectastain ABC kit (Vector Laboratory Inc., Burlingame, CA) according to the instructions of the manufacturer. Enzymatic development was performed in 0.05% diaminobenzene in PBS containing 0.003% hydrogen peroxide for 3–5 min. Quantification of GFAP-positive immunoreactivity was performed by counting the number of labeled cells per microscopic field using a 63x oil objective. For each brain, values from n = 5–10 striata were averaged to obtain a single value per mouse. Significant differences due to RGFP966 treatment were determined using Student’s t test (unpaired; two-tailed) (GraphPad Prism, San Diego, CA).

## Results

### Behavioral effects of RGFP966

**Fig 1:**
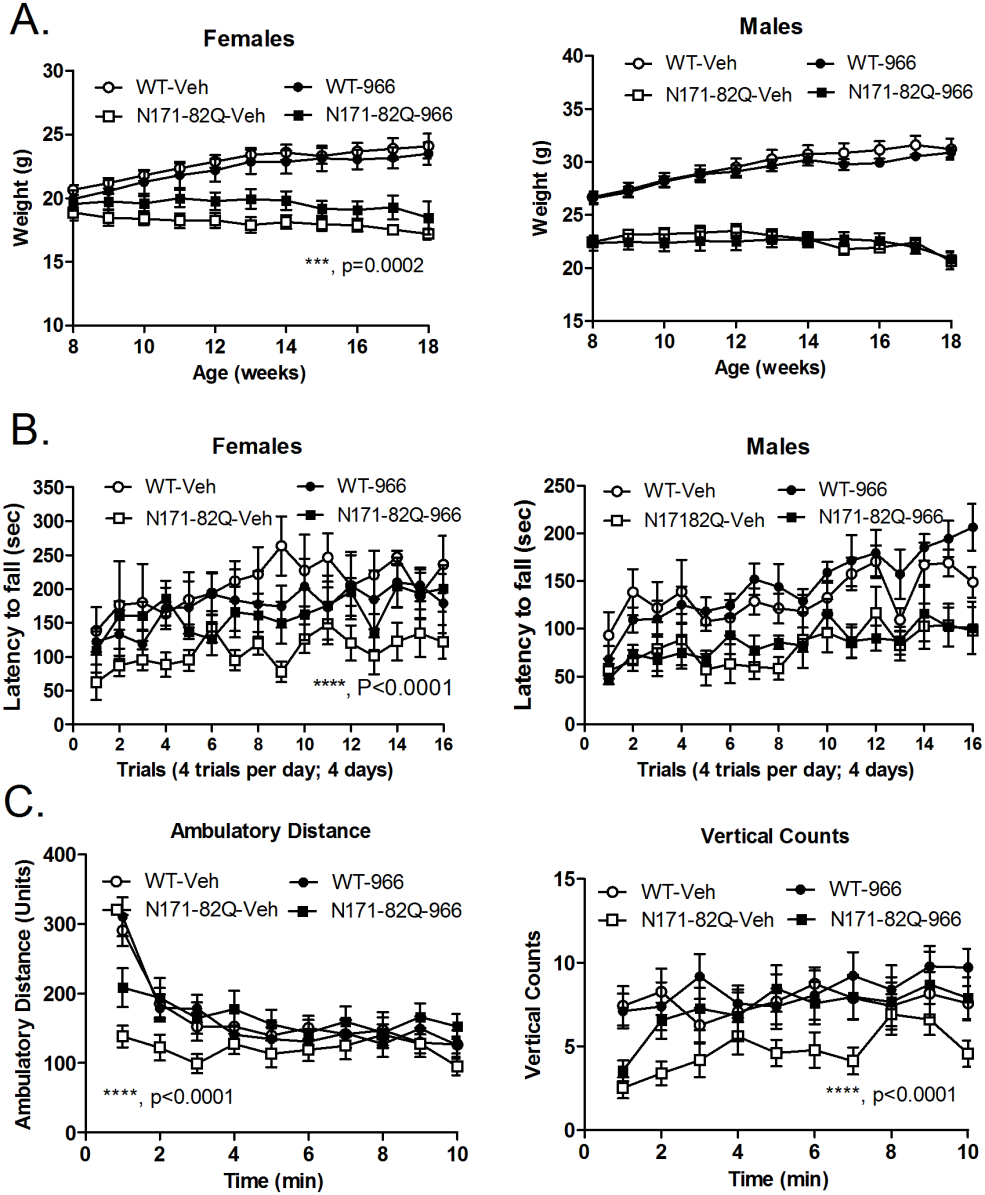
The behavioral effects of RGFP966 (25 mg/kg) on WT and N171-82Q transgenic mice. Panel A shows the effects of RGFP966 on the body weights of female and male WT and N171-82Q transgenic mice. Differences in drug- vs. vehicle-treated female mice were determined by Two-way ANOVA (***, F(1,122) = 5.6; P = 0.0002). Panel B shows rotarod performance of vehicle- and RGFP966-treated female and male N171-82Q transgenic mice. Two-way ANOVA revealed significant differences between drug- and vehicle-treated female transgenic mice (****, F(1,176) = 34.1; P<0.0001). (C) Effects of RGFP966 treatment on ambulatory distance and vertical counts in WT and N171-82Q transgenic mice over a 10 minute test period. Two-way ANOVA revealed significant differences between drug- and vehicle-treated N171-82Q transgenic mice in ambulatory distance (****, F(1,440) = 27.2; <0.0001), and vertical counts (****, F(1,450) = 23.5; P<0.0001). Additional measures of open field activity and the summary of effects of RGFP966 at different doses are shown in Table 1.

Here, we utilized the class I HDAC inhibitor, RGFP966, which shows a high degree of selectivity for HDAC3 over HDACs -1 and -2 (~30- and ~60-fold, respectively; S3 Table) with no activity detected at the other HDACs [20, 23], and low propensity to inhibit Cytochrome P450 activities (S3 Table). The distribution of RGFP966 to the CNS is relatively efficient, with a brain/plasma ratio of 0.43 (S3 Table), and as reported previously [20]. We have previously found that this compound shows improvement in motor function in HD mice at high doses (50 mg/kg) (Supp data from [24]). Here, we wanted to test the minimum effective dose of this compound, to optimize potential therapeutic applications. We tested the efficacy of two lower doses (10 and 25 mg/kg) in the N171-82Q transgenic HD mouse model. Mice were tested for motor dysfunction in the rotarod and open field activity tests, at 12 and 14 weeks of age, respectively. We found that RGFP966 treatment significantly improved body weight, rotarod performance and several measures of motor function in the open field locomoter test (Fig 1). A summary of results from both doses are provided in Table 1.

**Table 1:** Summary of the HD phenotypes affected by RGFP966 treatment at different doses in N171-82Q transgenic mice.

		RGFP966	
Test	Sex	25 mg/kg	10 mg/kg
**Body Weight**:	Male	NS	*p = 0.011
	Female	*** p = 0.0002	**p = 0.007
**Rotarod**	Male	NS	NS
	Female	**** p<0.0001	**** p<0.0001
**Open Field**:			
Ambulatory distance	Male & Female	**** p<0.0001	** p = 0.004
Ambulatory Time	Male & Female	** P = 0.0002	* p = 0.013
Resting Time	Male & Female	**** p<0.0001	* p = 0.042
Mean velocity	Male & Female	NS	NS
Vertical Counts	Male & Female	**** p<0.0001	NS
Vertical Time	Male & Female	** p = 0.0059	*p = 0.026

Statistical analyses for the different tests were carried out as described in Materials and Methods. NS, non-significant. In the Open field tests, there was no significant difference between the performance of male and female mice, hence, these data were combined.

### Neuroprotective effects of RGFP966

**Fig 2:**
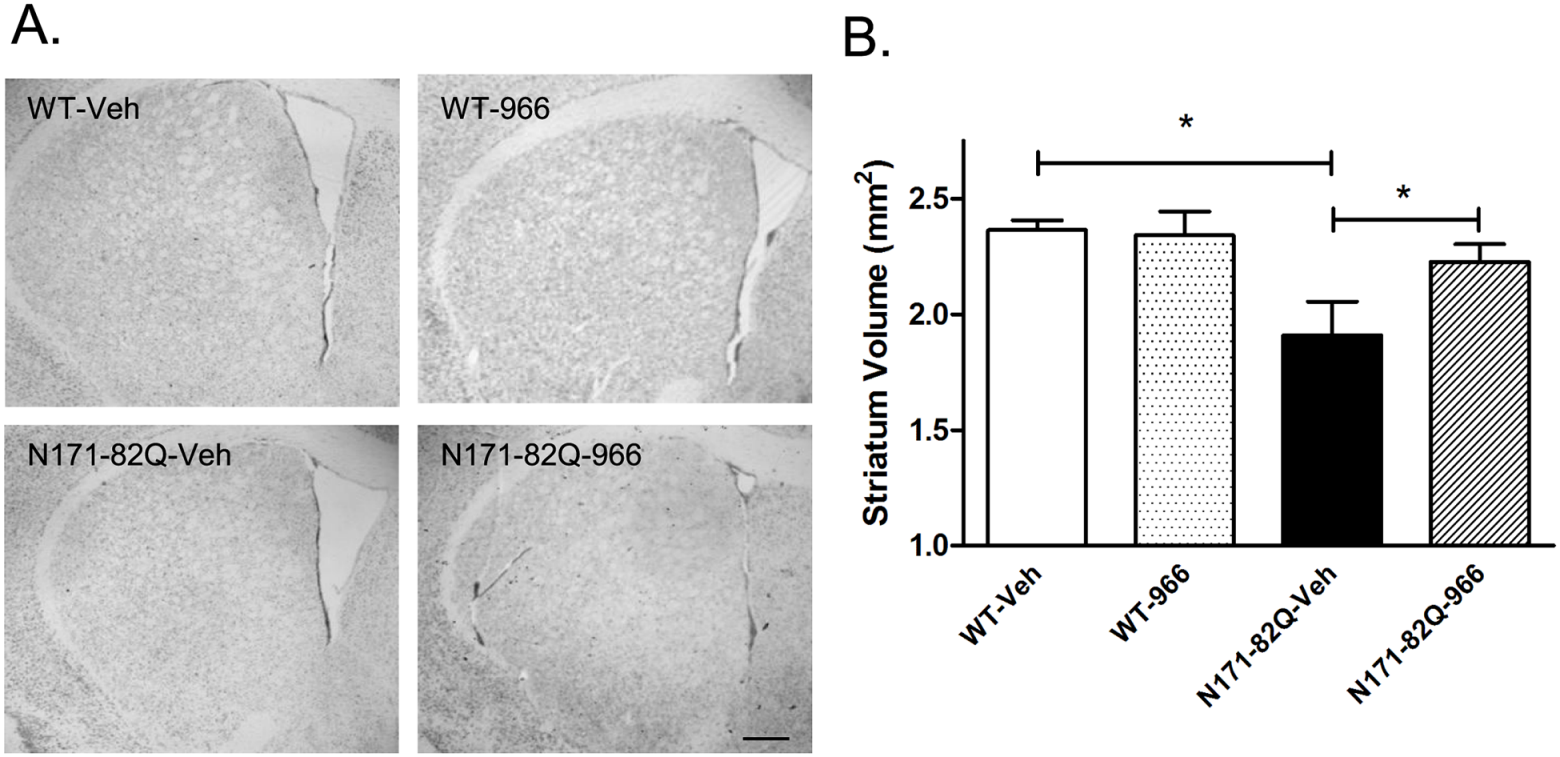
The effects of RGFP966 (25 mg/kg) on striatal volume in WT and N171-82Q transgenic mice. Representative photomicrographs of the striatum at ~0.62 Bregma under each condition are shown on the left. Scale bar = 40 μm. Bar graphs show quantitation of striatal volume. The total area of the striatum was assessed using sections 125 μm apart spanning the striatum from ~bregma, 1.18 to 0.38 mm. One-way ANOVA (Dunnett’s post-test) revealed significant differences between vehicle-treated wild type and N171-82Q transgenic mice and also a significant difference between vehicle-treated and RGFP966-treated N171-82Q mice (*, P<0.05). Bars represent mean score ± SEM (n = 6 to 7 per group).

We assessed the neuroprotective properties of RGFP966 by measuring striatal volume decline in N171-82Q transgenic mice that were treated at the 25 mg/kg dose, given that this dose was associated with more statistically significant effects on mouse behavior (see Table 1). Vehicle-treated N171-82Q transgenic mice showed a significant 19.3% decline in striatal volume, compared to WT littermate controls (One-way ANOVA; p<0.05). This effect was significantly prevented by chronic treatment with RGFP966, whereby RGFP966-treated mice showed only a 5.9% reduction compared to WT controls (Fig 2) (One-way ANOVA; p<0.05). However, RGFP966 did not cause a significant reduction of huntingtin aggregates in the brains of N171-82Q transgenic mice at 18 weeks of age, as measured by EM48 immunoreactivity (data not shown).

### Inflammation/cytokine gene expression

**Fig 3:**
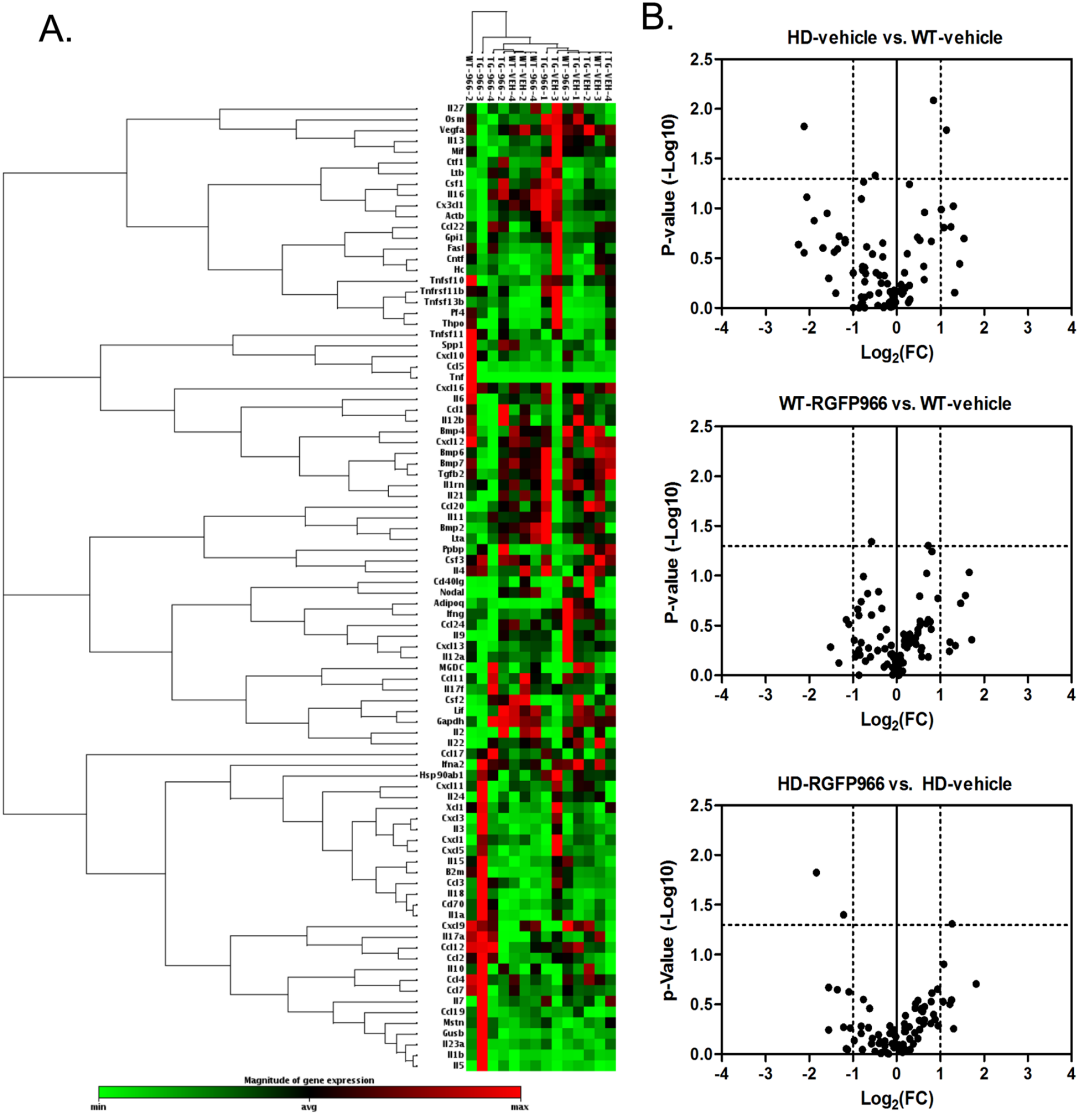
Summary of cytokines array gene expression changes in striatum due to RGFP966 treatment. A. Heatmap of expression values for 84 cytokine/chemokine genes showing two-way clustering of expression levels and treatment groups. Red denotes increased relative gene expression levels for the indicated groups, with green denoting decreased expression levels. B. Volcano plots of expression changes due to RGFP966 treatment showing three different comparisons, as indicated. Dotted line on y-axis denotes the significance cut-off of p-value<0.05, using one-way ANOVA. Dotted lines on x-axis denote a fold-change cut-off of > +/- 2.

Previous studies have demonstrated a role for HDAC3 in inflammatory gene expression pathways [10, 11]. Hence, we utilized a cytokine PCR array to test the effects of RGFP966 on immune-related gene expression in the striatum of N171-82Q transgenic mice at 18 weeks of age. The Cytokines & Chemokines PCR Array profiles the expression of 84 genes related to inflammatory and immune responses, including a range of interleukins, cytokines and chemokines (see S1 Table for gene list). Cluster analysis shows that, in general, the vehicle-treated N171-82Q group clustered separately from the RGFP-966 treated N171-82Q group and WT groups (Fig 3). Volcano plots show that, although several genes showed >2-fold changes in expression (log2 fold-change >1), the differences were not statistically significant for the comparisons show in Fig 3. Out of the 84 genes tested, only 3 showed statistically significant expression changes due to RGFP966 in N171-82Q striatum, suggesting that RGFP966 does not elicit a global change in immune signaling in the brain (Fig 3). The significant changes in expression (P<0.05) were observed in the following genes: chemokine (C-C motif) ligand 17 (*Ccl17*) increased 2.32-fold; macrophage migration inhibitory factor (*Mif*) decreased 2.4-fold; interleukin 13 (*Il13*) decreased -3.2-fold.

**Fig 4:**
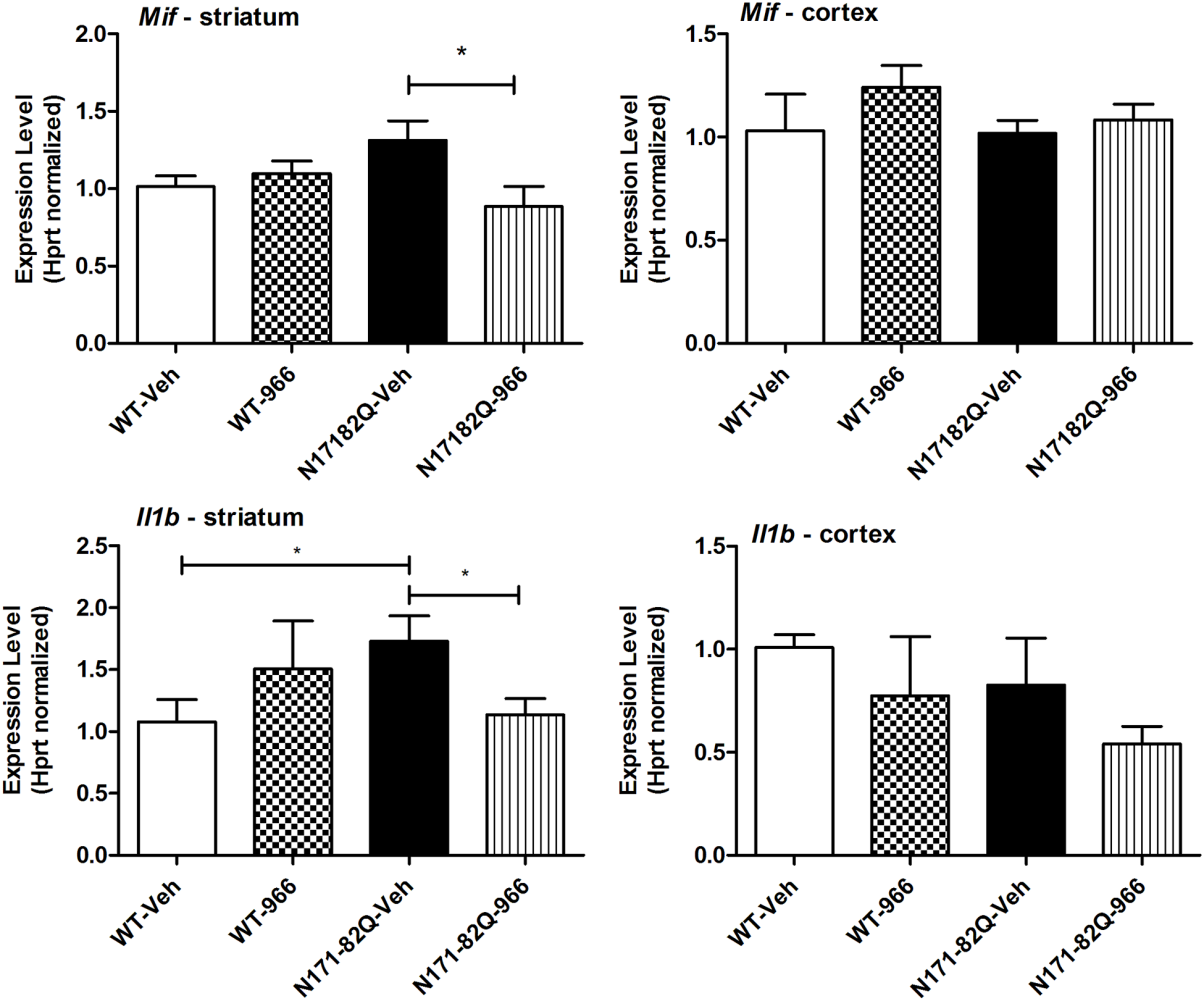
Real-time qPCR results showing altered expression of *Mif* and *Il1b* in striatum and cortex of RGFP966 treated WT and N171-82Q mice. Groups of mice were treated with RGFP966 (25 mg/kg) for 12 weeks beginning at 8 weeks of age. Bar graphs shown the mean +/- S.E.M. expression value from n = 5–6 mice per group normalized to the expression of *Hprt*. Significant differences of p<0.05 were measured by a two-tailed, unpaired Student’s *t* test and are indicated by an asterisk (*).

We further validated expression changes for one particular gene, *Mif*, which encodes a proinflammatory marker MIF, in both striatum and cortex, using primer sets different from those present on the PCR array. Results confirmed that RGFP966 caused a significant decrease in *Mif* expression within the striatum (Student’s t test; p<0.05) (Fig 4). A similar drug-induced decrease was not observed in the cortex (Fig 4). MIF has been shown to increase levels of other proinflammatory cytokines, encoded by Interleukin 1, beta (*Il1b*), Interleukin 6 (*Il6*) and Tumor necrosis factor (*Tnf)* [25]. These genes were found to be decreased in response to RGFP966 on the PCR arrays, but the differences did not reach statistical significance. Hence, we further validated expression changes for these genes by quantitative real-time PCR using distinct sets of primers from those used in the PCR array (see S2 Table). Decreased expression of *Il1b* in response to RGFP966 was apparent in the striatum (Student’s t test; p<0.05), but not the cortex of N171-82Q mice, mimicking the pattern observed for *Mif* in these regions (Fig 4). No significant changes in *Il6* or *Tnf* expression due to drug treatment were observed in either region (data not shown).

### GFAP immunoreactivity

**Fig 5:**
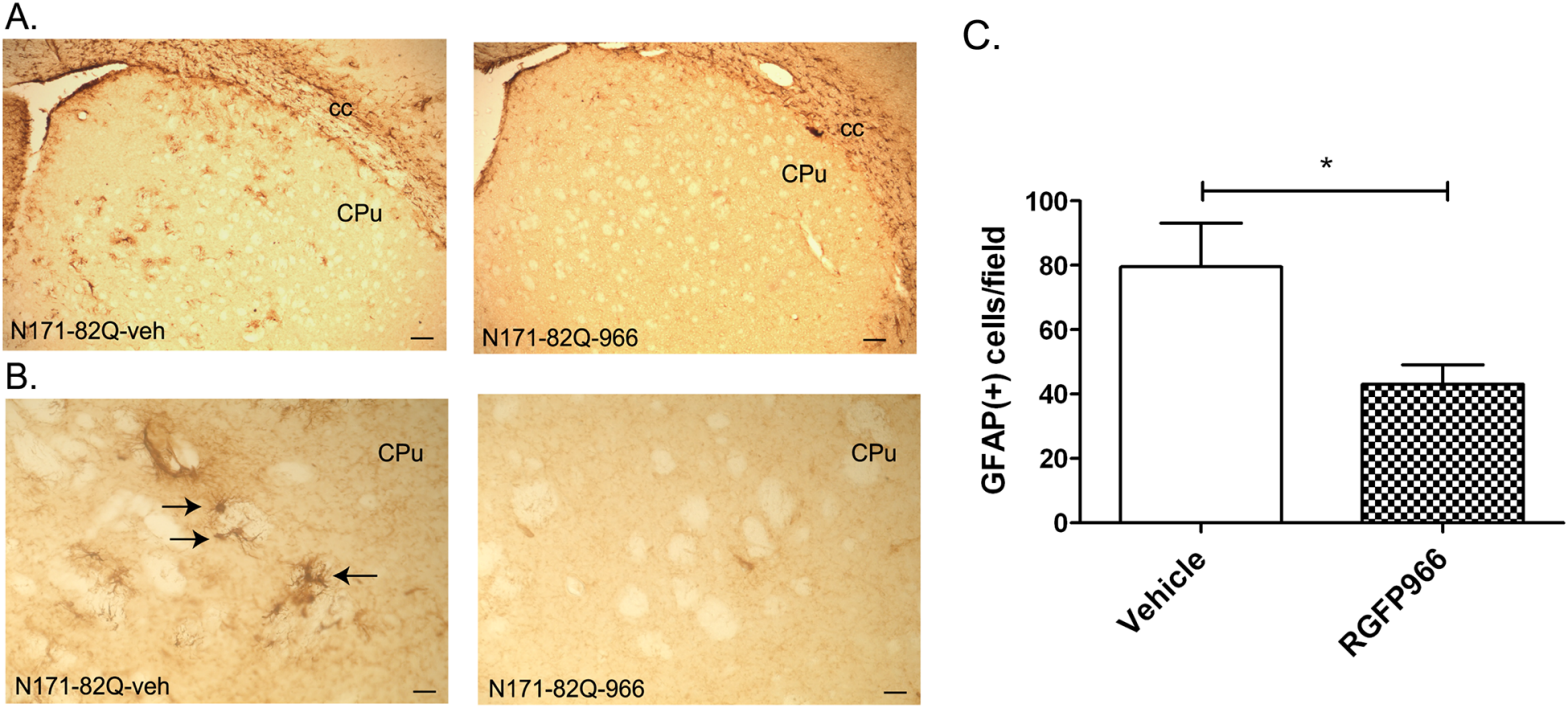
The effects of RGFP966 (25 mg/kg) on GFAP immunoreactivity in striatum of N171-82Q transgenic mice. Immunohistochemistry experiments were performed on free-floating brain sections (25 μm) from vehicle, and RGFP966-treated N171-82Q transgenic mice using an antibody to GFAP (Abcam; 1:500 dilution). A. Representative photomicrographs showing GFAP immunoreactivity in striatum. Caudate putamen, CPu; corpus callosum, cc. Scale bar = 20 μm. B. Arrows highlight GFAP (+) cells in the caudate putamen. Scale bar = 5 μm. C. Bar graph shows significant difference between vehicle-treated and RGFP966-treated N171-82Q mice (*, P<0.05; Student’s t test, unpaired, two-tailed). Bars represent mean score ± SEM (n = 6–7 per group).

Several studies have shown that *Mif* mRNA and protein are expressed in astrocytes of bovine, mouse and humans and elevated levels are associated with an inflammatory phenotype [26–28]. Given the reduction in expression of *Mif* by RGFP966, we sought to determine whether RGFP966 might affect glial activation by measuring glial fibrillary acidic protein (GFAP)-immunoreactive cells in the striatum of N171-82Q transgenic mice. In vehicle-treated N171-82Q transgenic mice, GFAP (+) cells could be observed in several regions of the brain, including the striatum at 18 weeks of age (Fig 5). In RGFP966-treated brains, there were fewer GFAP (+) cells in the striatum when compared to vehicle-treated animals (Fig 5), suggestive of lower astrocyte activation. Quantification of these cells in the striatum showed a significant 45% fewer number of GFAP (+) cells in RGFP966-treated animals (Student’s t test; p<0.05) (Fig 5).

## Discussion

We have previously shown that novel HDAC inhibitors preferentially targeting HDAC1 and HDAC3 can improve disease phenotypes in HD model systems [16–19]. Accumulating evidence has implicated these two subtypes in polyglutamine disorders [4]. Here we find that HDAC3 inhibition is sufficient to ameliorate disease symptoms in N171-82Q transgenic mice. One potential caveat is that RGFP966 can also inhibit HDAC1 at higher concentrations (S1 Table), however, given the low doses used in this study, we do not think HDAC1 contributes significantly to the effects of RGFP966 observed herein. HDAC3 has been specifically implicated in HD and even identified as a candidate genetic modifier based on information from the HD Research Crossroads database combined with genome-wide linkage studies [29]. Further, Bardai and colleagues found that WT huntingtin protein, but not the mutant form, interacts with HDAC3. According to this model, the expression of mutant huntingtin liberates HDAC3 from the normal form, thus removing the natural repression of its neurotoxic actions [9]. A reduction in huntingtin-HDAC3 interaction is also seen in neurons exposed to other apoptotic stimuli and in the striatum of R6/2 HD mice [9].

Altered expression of HDAC3 has also been shown in HD mouse models. We found elevated HDAC3 mRNA levels in striatum of late-stage 18 week old N171-82Q transgenic mice (S1 Fig), which was similar to our previous studies showing elevated HDAC1 and HDAC3 protein levels in nuclear fractions from within the cortex of late-stage N171-82Q transgenic mice [16]. However, although expression levels of HDAC3 were not found to be significantly different in cortical samples from R6/2 transgenic mice in a different study, the striatum did show a non-significant increases in protein expression at 9 weeks of age [30].

Genetic reduction of HDAC3 using *Hdac3 (+/−)* heterozygous mice has been reported, and was not found to ameliorate disease phenotypes when crossed with R6/2 transgenic mice [31]. However, in that study, the overall protein levels of HDAC3 were only reduced by 20% in the heterozygous double mutant animals [31], hence it is likely that the these reductions were not sufficient to observe significant changes in HD phenotypes.

Of interest to our group is what mechanism(s) is/are associated with the preclinical efficacy of pharmacological inhibition of HDAC3. Although our cytokine profiling array did not reveal widespread alterations to critical proinflammatory cytokines, it did reveal a few distinct changes. The most notable change was observed in the expression of *Mif*, whose protein product MIF has been identified as a hormone and immunomodulator, as well as a multipotent cytokine that exerts a variety of mitogenic and proinflammatory functions. MIF has been shown to promote microglial activation and production of the innate soluble mediators IL1β, IL6, TNFα, and inducible nitric oxide synthase in primary microglial cells from C57BL/6 neonatal mice. [25]. Additional qPCR assays found that RGFP966 treatment also caused a reduction of *Il1b* in the striatum, with no observable changes in the cortex, similar to the effect of the drug on *Mif* expression. This finding is consistent with previous studies showing that MS-275 and SAHA, two potent but non-selective HDAC inhibitors, suppressed the expression of MIF in human rheumatoid arthritic synovial fibroblastic E11 cells [32].

Because HDAC3 inhibition elicited a decrease in the expression of *Mif*, one might predict that the mechanism of action for this effect does not involve altered histone acetylation, which would be expected to result in increased gene expression. However, several non-histone transcription factor targets have been identified for HDAC3, including SMAD Family Member 7 Myocyte enhancer factor 2, Mitogen-activated protein kinase phosphatase-1 (MKP-1) and nuclear factor-kappaB (NFkB) [33–36]. Interestingly, two of these, MKP-1 and NFkB, have been specifically implicated in inflammation [36].

MIF is expressed within astrocytes in the brains of different species, including humans [26–28] and has been associated with astrocyte activation. Intracerebroventricular injection of streptozotocin was found to increase astrocyte activation and MIF expression in the hippocampus of APP/PS1 transgenic mice, whereby *Mif* deficiency was found to attenuate astrocyte activation in this model [37]. Consistent with these results, we found that the RGFP966-induced decrease in *Mif* expression in N171-82Q transgenic mice was associated with fewer GFAP (+) cells in the striatum when compared to vehicle-treated animals. We hypothesize that this effect might be mediated via a reduction of *Mif* expression specifically in this region, eliciting a downstream decrease in astrocyte activation.

We did not observe decreases in the levels of huntingtin aggregates in the striatum of N171-82 transgenic mice in response to HDAC3 inhibition. This is consistent with previous studies using non-selective HDAC inhibitors [38, 39], but in contrast to our previous studies using an HDAC1/3-targeting compound, HDACi 4b, where fewer EM48-(+) aggregates were detected in the cortex of HD mice treated with this drug [16]. These findings might suggest that HDAC1 inhibition may play a prominent role in reducing huntingtin aggregation. This idea would support prior work showing that knock-down of HDAC1 in Htt590-97Q-transfected Neuro2a cells leads to specific increases in acetylation of lysine 444 of the huntingtin protein, promoting its degradation by the autophagy pathway [40]. However, a recent study using HeLa cell lines expressing huntingtin exon-1, has also implicated HDAC3 in controlling huntingtin levels (Mano et al. 2014). In that study, it was found that treatment with the HDAC3-selective inhibitors, T247, T326 and T130, caused a decrease in cytoplasmic levels of soluble huntingtin as measured by Western blot, but an elevation of insoluble huntingtin protein in the nucleus as measured by filter-trap assays (Mano et al. 2014). We did not distinguish between the location (cytoplasmic vs. nuclear) or the size of the huntingtin aggregates in our study, which may account, in part, for the lack of effect we observed due to RGFP966 treatment. Alternatively, it is likely that the aggregation pathways in non-neuronal cultured cells differ from those in the N171-82Q transgenic mouse brain.

Building on these findings, and those from available literature, we suggest that inhibition of HDAC3 has potential therapeutic applications for the treatment of HD in humans. However, these studies do not rule out a role for other HDAC subtypes in the pathology or therapeutics of HD.

## Supporting Information

S1 Methods Ethical considerations for the use of N171-82Q transgenic mice in our study. (DOCX)

S1 Table List of genes on the cytokine PCR array, PAMM150Z. (XLSX)

S2 Table Primer sequences used for qPCR analysis. (DOCX)

S3 Table HDAC subtype selectivity profiles for RGFP966. (XLSX)

S1 Fig Real-time qPCR results showing elevated expression of HDAC1 and HDAC3 in striatum of N171-82Q transgenic mice compared to WT controls. (TIF)

## Abbreviations

HD: Huntington’s disease
Htt: huntingtin
HDAC: histone deacetylase
S.C.: subcutaneous
C-C motif: chemokine
Ccl17: ligand 17
Mif: macrophage migration inhibitory factor
Il13: interleukin 13
Il1b: interleukin 1, beta
Il6: interleukin 6
Tnf: tumor necrosis factor
