# Supplementary material for: The Effects of Pharmacological Inhibition of Histone Deacetylase 3 (HDAC3) in Huntington’s Disease Mice
Source: PLoS One. 2016 Mar 31;11(3):e0152498. doi: 10.1371/journal.pone.0152498 (PMC4816519; doi:10.1371/journal.pone.0152498)
Supplement: S1 Methods — (DOCX) [file pone.0152498.s001.docx]

**Ethical considerations for the use of N171-82Q transgenic mice in our study.**

All procedures were in strict accordance with the National Institutes of Health Guidelines for the Care and Use of Laboratory Animals and were approved by the Scripps Research Institute’s Institutional Animal Care and Use Committee. All procedures are designed to cause only momentary or no pain to the animals. HD N171-82Q transgenic mice in this study may suffer mild distress due to disease progression (motor dysfunction & weight loss). This type of distress is unavoidable, as it is the nature of this disease model. N171-82Q transgenic mice develop symptoms between 8 and 10 weeks of age, with death occurring typically by 5 months of age. Symptoms include motor impairments, such as loss of coordination and slowness of walking, as measured by rotorod testing or open field activity monitoring. Criteria for early removal of debilitated animals include monitoring rotarod behavior. If a mouse cannot stay on the rotarod at least 20 seconds after day 1 of training, then the mouse is euthanazied. Mice also display weight loss of about 10-15% of body weight near end stages (i.e. ~4 months of age). HD transgenic mice are monitored daily at the ages of >4 months and euthanized if weight loss is greater than 20% adult body weight. Further, if a mouse develops early symptoms of disease, such as lack of grooming or hunched posture, at less than 2 months of age, the mouse will be euthanized.
