# Supplementary material for: The Effects of Pharmacological Inhibition of Histone Deacetylase 3 (HDAC3) in Huntington’s Disease Mice
Source: PLoS One. 2016 Mar 31;11(3):e0152498. doi: 10.1371/journal.pone.0152498 (PMC4816519; doi:10.1371/journal.pone.0152498)
Supplement: S2 Table — (DOCX) [file pone.0152498.s003.docx]

**Supplementary Table 2. Primer Sequences used for qPCR Analysis.**

Mif-F: 5’-ACGACATGAACGCTGCCAAC-3’

Mif-R: 5’-AGGCTCAAAGAACAGCGGTG-3’

Il1b-F: 5’-GGTACATCAGCACCTCACAAG-3’

Il1b-R: 5’-GCCCATACTTTAGGAAGACACG-3’

Hprt-F: 5’-TGATGAAGGAGATGGGAGGCCA-3’

Hprt-R: 5’-CCAGCAGGTCAGCAAAGAACTTAT-3’
