# Supplementary figures and images for: The Effects of Pharmacological Inhibition of Histone Deacetylase 3 (HDAC3) in Huntington’s Disease Mice
Source: PLoS One. 2016 Mar 31;11(3):e0152498. doi: 10.1371/journal.pone.0152498 (PMC4816519; doi:10.1371/journal.pone.0152498)

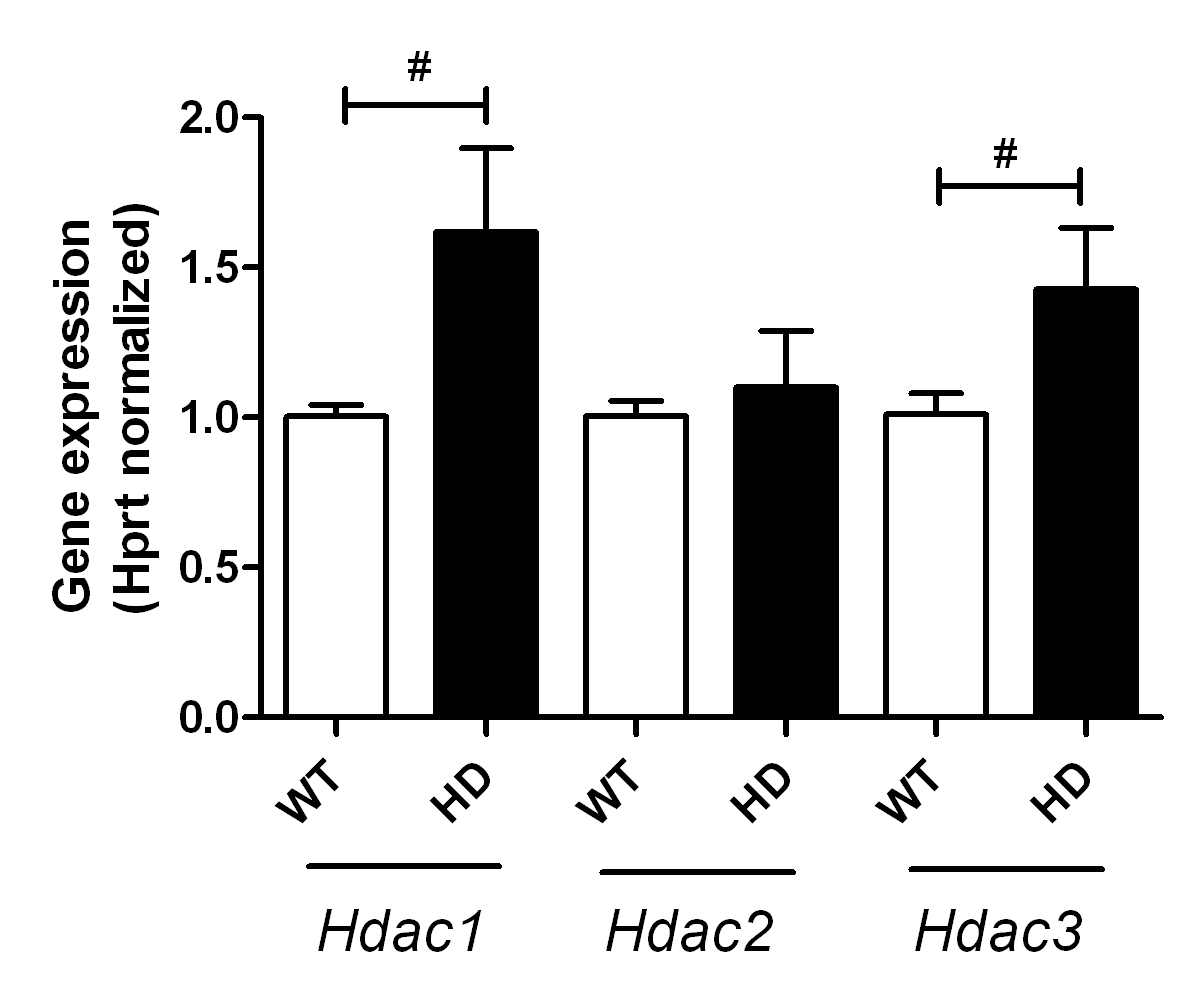

Supplement: S1 Fig — (TIF) [file pone.0152498.s005.tif]
